# Effect of eccentric isokinetic strengthening in the rehabilitation of patients with knee osteoarthritis: Isogo, a randomized trial

**DOI:** 10.1186/1745-6215-15-106

**Published:** 2014-04-02

**Authors:** Anne-Gaëlle Jegu, Bruno Pereira, Nicolas Andant, Emmanuel Coudeyre

**Affiliations:** 1 CHU Réunion, Service de Médecine Physique et Réadaptation, Site du Tampon, GHSR Saint-Pierre, Saint Pierre BP 350-97448, France; 2 CHU Clermont-Ferrand, Biostatistics Unit, Délégation Recherche Clinique & Innovation (DRCI), Villa annexe IFSI, Clermont-Ferrand F-63003, France; 3 Service de Médecine Physique et de Réadaptation, Clermont University, Université d’Auvergne, F-63000 Clermont-Ferrand; CHU Clermont-Ferrand, 58, rue de Montalembert, Clermont-Ferrand F-63000, France

**Keywords:** Eccentric, strength, isokinetic, knee osteoarthritis, rehabilitation

## Abstract

**Background:**

Femorotibial knee osteoarthritis is associated with muscle weakness in the lower limbs, particularly in the quadriceps, which results in disease progression. The interest of having muscular strengthening as part of the therapeutic arsenal for the medical treatment of knee osteoarthritis is now well established.

The functional disability induced by knee osteoarthritis manifests itself principally when walking, notably downhill, during which the muscles are called upon to contract eccentrically.

We can therefore think that eccentric muscular strengthening could bring a functional benefit that is superior to concentric muscular strengthening.

**Methods/Design:**

This is a prospective, randomized, bicenter, parallel-group, international study. Eighty patients aged from 40 to 75 years old, suffering from medical-stage knee osteoarthritis, will undertake 6 weeks of isokinetic muscular strengthening. Randomization determines the mode of muscular strengthening: either exclusively eccentric or exclusively concentric.

The principal objective is to demonstrate the superiority of the improvement in the quadriceps isokinetic torque after isokinetic muscular strengthening by the eccentric mode compared to the concentric mode.

The following parameters are also evaluated: the variations in the level of pain, the parameters of walking (maximum speed over 10 and 200 meters, analysis on a computerized Gaitrite™ treadmill), static equilibrium (on a FUSYO™ force platform), and the functional status of the patient using the Western Ontario and MacMaster Universities osteoarthritis index (WOMAC) questionnaire after the strengthening period and at 6 months.

**Discussion:**

A better knowledge of the most effective mode of muscular strengthening is needed to optimize the functional benefits to the patients. In case of superiority in terms of efficacy of the eccentric mode, the latter could be given priority in the rehabilitation treatment of knee osteoarthritis patients.

**Trial registration:**

Clinical trials.gov number: NCT01586130.

## Background

By 2020, the increase in life expectancy and the aging of the population should bring arthritis to fourth place in the causes of disability in developed countries [1].

The knee is one of the most frequent arthrosic locations. In 2002, it represented 1.7% of the health insurance expenses in France [2]. Its therapeutic care relies on the association of pharmacological and nonpharmacological treatments [3].

Patients suffering from knee osteoarthritis present a muscular weakness in the legs, particularly the quadriceps [4-6]. The extent of quadricep muscle deficiency is linked to the level of gonalgia and functional disability [7]. It has been proven that physical activity and muscular strengthening improves these two factors. On the other hand, we do not know which mode of muscular strengthening is the most efficient. The current recommendations suggest an adapted and personalized rehabilitation program concerning the type of exercises, the intensity, and the frequency [8]. Several studies have shown the interest of isokinetic dynamometers as rehabilitation tools in muscular strengthening and have found an equivalent or superior efficacy of this rehabilitation mode compared to the isometric or isotonic physical exercises more commonly used in current practice [9,10]. The isokinetic exercises also present an advantage in terms of cardiac tolerance, which is interesting for aged, potentially vascular, arthrosic patients. Indeed, it leads to a smaller increase in heart rate and blood pressure than isometric exercises [11].

The eccentric contraction of the quadriceps muscles seems to play a fundamental role in walking and other activities of everyday life, allowing control of the bending of the knee (cushioning) and an active joint stability [12]. The protocols for isokinetic muscular strengthening, in combined concentric-eccentric mode, have shown better results than the concentric mode alone in terms of functional improvement in knee osteoarthritis [13].

To our knowledge no published study has compared isolated eccentric strengthening to concentric strengthening alone in knee osteoarthritis (OA) whereas the superiority of eccentric strengthening in terms of increase in strength has been demonstrated elsewhere for other pathologies [14]. Thus, rehabilitation care currently practiced could be optimized by preferentially using this muscular strengthening contraction mode.

## Methods/Design

### Study participants

Patients will be recruited during consultations in Physical and Rehabilitation Medicine (PRM) and in Rheumatology in the hospital or in private practice. Patients’ rehabilitation histories and osteoarthritis therapies will be collected to fully appreciate possible cofounding biases. The selection criteria for patients for the ISOGO study are shown in Table 1.

**Table 1:** Inclusion and exclusion criteria of ISOGO study

**Inclusion criteria**	Aged between 40 and 75 years old
	Knee osteoarthritis according to the criteria of the American College of Rheumatology
	Unilateral medial femorotibial knee osteoarthritis
	No corticosteroid or hyaluronic acid infiltration within the 2 months before inclusion
**Exclusion criteria**	Knee arthroplasty
	Radiological end-stage knee osteoarthritis: stage 4 of Kellgren and Lawrence classification
	Associated symptomatic femoropatellar osteoarthritis
	Language or mental issues preventing the understanding of the protocol
	Pregnancy

Patient eligibility will be defined as symptomatic knee OA in the medial compartment, fulfilling the American College of Rheumatology (ACR) clinical criteria for OA of the knee. Patients will be included at least 2 months after corticosteroid or hyaluronic acid injection.

Radiographs will be scored using the Kellgren Lawrence (KL) global classification, which assigns a number from 0 (normal) to 4 (severe) based on the presence of osteophytes, joint space narrowing, sclerosis and joint deformity. Patients with grade 4 KL radiological classification will be excluded from the study.

Authorization was obtained from the institute’s committee on human research (CPP Sud Est VI) in September 2011. Prior to enrollment in the study, all subjects will be asked to give their informed consent. Authorization from the AFFSAPS was obtained in September 2011. The study is registered with Clinical trials.gov under the number: NCT01586130.

### Study design

**Figure 1:**
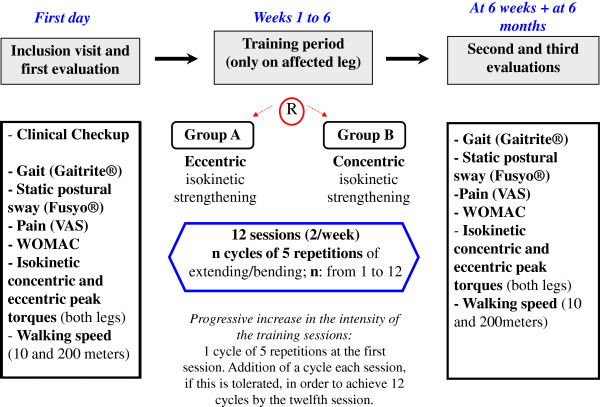
Study design: This flow-chart (to be read from left to right) displays the different phases of the protocol and explorations being conducted.

ISOGO is a prospective randomized double center clinical trial with blinded evaluation of the endpoints. The outline of the study is shown in Figure 1.

#### Randomization

Included patients will be randomly allocated to either arm (training group) in a 1:1 ratio. The randomization list will be generated by block randomization. Randomization will be performed automatically (using software) by an independent statistician once a patient will be considered eligible and informed consent will be obtained.

#### Isokinetic training

All tests and training sessions will be completed on the HumacNorm Cybex 6000™ computer-controlled isokinetic dynamometer, which will be calibrated before every test.

Patients with medial femorotibial knee osteoarthritis will be recruited and randomized into two groups of 40:

1. Group A: (eccentric isokinetic training): 12 sessions of eccentric isokinetic extension-flexion repetitions of the affected knee.

2. Group B: (concentric isokinetic training): 12 sessions of concentric isokinetic knee extension-flexion repetitions of the affected knee.

Patients will have resistance training two times a week for 6 weeks (12 sessions) with a minimum of 1 day of rest between sessions in order to minimize the potential effect of delayed onset muscle soreness (DOMS).

Participants will begin each session with a 5-minute warm-up on a stationary cycle ergometer at low rate and low load. After warm-up, patients’ will complete extensor and flexor stretches under the supervision of the physiotherapist. The affected knee then will receive the rehabilitation treatment. The first session will consist of one set of five repetitions of reciprocal concentric or eccentric isokinetic knee extensions (according to randomization) and flexion with maximal effort. The number of sets of repetitions will progressively increased to achieve 12 sets of five repetitions on the last session.

To monitor progress and limit habituation to a specific contraction mode, the seventh session will be preceded by a repeat of the isokinetic testing evaluation completed at the baseline (testing on both the eccentric and concentric modes and on both knees).

### Objectives and endpoints

The primary objective of this study will be to assess whether eccentric isokinetic strengthening improves the isokinetic strength of the quadriceps more than concentric isokinetic strengthening in patients with medial femorotibial knee osteoarthritis. The concentric isokinetic torque of the quadriceps will be used as primary outcome measure because of its better reliability and reproducibility compared to an eccentric isokinetic torque [15].

The secondary objectives will be to compare the effects of concentric and eccentric isokinetic training on:

1. Static postural balance on a Fusyo™ force platform

2. Temporospatial gait parameters assessed by Gaitrite™

3. Maximal 10 meters and 200 meters at walking speed

4. Range of knee motion

5. Pain, stiffness and function assessed by the Western Ontario and MacMaster Universities osteoarthritis index (WOMAC) questionnaire.

These access joint mobility but also physical function in daily living activities, which influence improved feeling for the most patients. The effects of 6 weeks of training on these parameters will be evaluated after the 12 strengthening sessions and at 6 months. The possible adverse effects of the different strengthening modes will also be analyzed (muscular and articular pain evaluation before and after each session, maximal cardiac frequency during sessions, and muscular lesions).

### Assessments

#### Isokinetic tests

Subjects will seat with the backrest at a 90° angle and instructed to grip the sides of the seat during the test. The thigh, pelvis, and trunk will be stabilized with straps. An adjustable lever arm will be attached to the subject’s leg by a padded cuff just proximal to the lateral malleolus. The axis of rotation of the dynamometer arm will be positioned just lateral to the lateral femoral epicondyle.

Conventional concentric and eccentric continuous isokinetic tests will be used. During concentric and eccentric tests, the subjects will continuously push the lever arm of the isokinetic device up and down through the whole range of motion between 10° and 90° (0°: straight leg) to measure quadriceps and hamstring strength. Subjects will not systematically have prior experience with the isokinetic dynamometer and will familiarize themselves with testing procedures by performing three consecutive warm-up trials for each muscle group and speed.

During tests the subjects will perform five maximal continuous flexion-extension repetitions of both legs at each angular velocity: 60°/s and then 180°/s in concentric mode and 30°/s in eccentric mode (according to the randomization). During the concentric tests the speed order will be from slower to faster, as suggested by Wilhite *et al*. [16]. A 1-minute rest will be allowed between each contraction speed. Eccentric tests will be performed after the concentric tests. A 5-minute rest will be allowed between legs. The researcher conducting the isokinetic evaluation will be blinded to the strengthening mode received by the patient. The subjects will be verbally encouraged to exert maximal effort.

#### Clinical examination at inclusion

The following data will be recorded:

1. Knee medical history;

2. Range of motion of the knee;

3. Thigh circumference at 5, 10 and 15 cm from the base of the patella;

4. Blood pressure,

5. Heart rate;

6. Anthropometric measurements (height, weight, body mass index); and

7. Electrocardiogram.

#### Western Ontario and MacMaster Universities osteoarthritis index self-questionnaire

Patients’ pain, stiffness and physical function will be assessed using the administered WOMAC questionnaire. This activity-based questionnaire includes 24 questions (five on pain, two on stiffness and 17 on physical function disability) and has been shown to be a reliable and valid multidimensional health status instrument in knee and hip OA.

#### Measurements of postural sway

Static postural sway will be assessed using a force platform (Fusyo™) under four conditions, in the following order:

1. Bipedal stance with eyes open;

2. Bipedal stance with eyes closed;

3. Monopedal stance on nonaffected leg, with eyes open; and

4. Monopedal stance on affected leg, with eyes open.

The first condition will be performed twice: the first time to acclimatize the subject and the second time for the measurement. The data will be acquired at 40 Hz over a period of 52 seconds, in accordance to the ‘Normes 85’ of the French Association of Posture and Equilibrium [17].

During all balance tests, subjects will be told to look straight ahead. During the bipedal stance, subjects will stand barefooted on the force platform with their feet positioned at shoulder width and their arms by their sides. For each subject, the positions of the footplates will be adjusted to match the subject’s normal stance. During the monopedal, stance subjects will be instructed to hold their contralateral limb at 90° of knee flexion for the duration of the test. Before starting the test and data collection, the subjects will be allowed to settle into a good test position. When the subjects will be ready they will be asked to say ‘ready’ and data collection will start. The subjects will be allowed to practice each test condition a few times before the actual measurements will be taken.

#### Spatiotemporal parameters

A 7.93-meter-long computerized treadmill (Gaitrite™, CIR Systems Inc.) will be used. Patients will be instructed to walk barefoot at a self-selected speed as previous studies have shown that this gives a consistent result for the gait parameter [18]. Each evaluation will include four comings and goings. The mean value of the eight tests will be analyzed for each parameter.

The following spatiotemporal parameters will be evaluated: absolute velocity (m/s), normalized velocity (m/s/leg length), cadence (steps/min), step length (m), normalized step length (m/leg length), swing phase (% gait cycle), stance phase (% gait cycle), single limb support phase (% of gait cycle), double limb support phase (% gait cycle), and base of support (m).

### Statistical considerations

#### Justification of the number of subjects to be included

The principal objective of this study lies in the assessment of the increase in the quadriceps isokinetic peak torque of the treated knee as measured using a Cybex Norm™ computerized dynamometer, with the concentric mode, at a speed of 60/second after treatment.

The estimation of sample size was determined on the basis of a greater improvement of muscular strength in the quadriceps of the treated knee (muscular strengthening in the medical treatment of the femorotibial knee osteoarthritis) in the eccentric group compared to the concentric group. From the work of Tüzun *et al.*[10] and Huang *et al*. [9], we learned about the increase in muscular strength of the quadriceps using isokinetic concentric training. The difference between the two groups was estimated at 15% (treated as quantitative) and the standard deviation was fixed at 20 (supposedly identical in the two groups). Also, for a type I error α and a statistical power fixed respectively at 5% (in a two-sided situation) and 90%, 40 patients per group need to be recruited. The rate of ‘lost to view’ for the measurement of the principal endpoint criterion at 6 weeks is considered negligible [10].

#### Principal analysis

The principal endpoint criterion is the improvement at 6 weeks of treatment of the isokinetic concentric quadriceps peak torque at the speed of 60°/s. The comparison between the randomized groups will be performed using the Student test or the Mann and Whitney test (if the conditions for validity of the Student test are not respected). The comparison between the treatment groups will be performed systematically: 1) without adjustment and, 2) by adjusting other factors whose repartition could be, despite the randomization, unbalanced between the treatment groups.

#### Secondary analysis

The analyses for the secondary endpoints will be performed in a similar way to those previously described. The same applies to the secondary endpoint criteria: pain, functional status by WOMAC, maximal walking speed and performance, measurement of peak torques. The comparison of the qualitative parameters (for example the WOMAC scale categorized, for example, by median or quartiles) between the two groups will be made using the Chi-square test or Fisher's exact test (if appropriate).

In order to measure the progression of the different parameters collected during the three visits (mobility, amyotrophy, deformation, effusion, and pain on palpation), we will conduct a longitudinal analysis of the data in two stages by ANOVA for repeated data followed by a post-hoc Tukey-Kramer test and using mixed models (or random effects models) [19] that will allow the measurement of the intrasubject correlation by taking into account the effects of the subject and time (‘random intercept’ and ‘slope’ effects). An associated measurement of the coefficients of intraclass correlation will also be performed. The impact of covariables will be also explored.

#### Method of accounting for missing, unused or invalid data

A sensitivity analysis of missing data will be performed to ensure the pertinence of the longitudinal data (MAR or MCAR). In order to assess the problem caused by missing longitudinal data at 6 months, estimation methods developed by Verbeke and Molenberg should be proposed [20,21].

## Discussion

In addition to being related to pain and a limitation in functional capacity, muscle weakness influences the progression of OA [7,22]. Severe levels of disability or inactivity induce muscle wasting and may contribute to the decline in strength reported in elderly people. These findings may have important implications for people with knee OA and knee pain, particularly in a therapeutic and preventative role of increasing muscle strength to reduce or stop disease progression. Scientific literature confirms that exercises, particularly strengthening exercises, are beneficial in terms of improved physical function and strength in people with knee osteoarthritis [23]. Yet, there is no scientific evidence for which type of strengthening exercises would have an impact on functional outcomes.

Isokinetic evaluation is used to quantify muscle strength, treatment and rehabilitation efficacy in case of mechanical [15] or neurological instability of the knee [24]. Moreover, isokinetic exercises can be used as personalized muscle strengthening techniques, offering a graduated and secure program with an objective measurement of the progress. Isokinetic exercises offer great selectivity for the motion required in ambulation and produce a faster rate of strength gain and reduced muscle tenderness than isotonic training [25]. Also, increases in heart rate and blood pressure seem to be lower with isokinetic compared to isometric exercises, which could be of particular importance among older patients with OA who often present comorbidities [11]. Interestingly, isokinetic eccentric exercises induce lower metabolic and cardiovascular responses than concentric exercises [26].

In accordance with the data in the literature, we expect to find an improvement of the quadriceps strength in the treated arm of the two groups after isokinetic strengthening. In case of superiority of the eccentric mode, the priority could be given to an eccentric training in the rehabilitation treatment of knee osteoarthritis patients.

### Trial status

The study has been in an active recruiting phase since April 2012.

## Abbreviations

ACR: American College of Rheumatology; DOMS: delayed onset muscle soreness; KL: Kellgren and Lawrence's classification; M: meters; Min: minutes; OA: osteoarthritis; S: seconds; WOMAC: Western Ontario and MacMaster Universities osteoarthritis index.

## Competing interests

The authors declare that they have no competing interests.

## Authors’ contribution

AGJ: Study design, data collection and analysis, manuscript writing and approval of the final manuscript. BP: Data analysis, critical revision and approval of final manuscript. EC: Study design, critical revision and approval of final manuscript. NA: Study setup, critical revision and approval of the final manuscript. All authors read and approved the final manuscript.
